# Exploratory integrative machine learning analysis of miRNA datasets identifies a candidate miRNA signature for oral squamous cell carcinoma

**DOI:** 10.1186/s44342-026-00090-7

**Published:** 2026-11-06

**Authors:** Rida Naveed, Hafiz Ghulam Murtaza Saleem, Safia Firdous, Muhammad Sohail, Qainaat Faiz, Iman Asif, Moeez Khalid

**Affiliations:** Department of Medical Lab Sciences, Riphah International University, Lahore, Pakistan

**Keywords:** Oral squamous cell carcinoma, Circulating microRNA, Machine learning, Biomarkers, GEO, TCGA

## Abstract

**Background:**

Oral squamous cell carcinoma (OSCC) is typically diagnosed at an advanced stage and thus there is a need for biomarkers for OSCC which can be detected with minimum invasiveness. Although circulating microRNAs (miRNAs) have demonstrated great diagnostic promise, there are limited publicly available circulating miRNA datasets associated with OSCC.

**Objective:**

To exploratively integrate public tissue miRNA datasets using machine learning methods to identify candidate miRNAs as potential OSCC biomarkers.

**Methods:**

The miRNA profiling of multiple OSCC tissue datasets from GEO and TCGA containing both tumor and control samples were integrated for biomarker discovery. Tumor samples only were excluded prior to the model development. Machine learning models were developed using differentially expressed set of miRNAs. Five classifiers were evaluated and the best model was externally validated by applying it to the only publicly available independent circulating serum miRNA dataset. The signature was subjected to a continuous probability score. The model interpretation was performed using SHAP.

**Results:**

A 30-miRNA candidate signature associated with OSCC was identified. In internal cross-validation, the panel separated tumor from normal tissue (AUC 0.96). The logistic regression model had the highest AUC, while XGBoost and SVM-RBF had slightly higher accuracy (Table 5) and gave an AUC of 0.67 in external validation. The held-out tissue-level performance was variable and lower (AUC 0.54–0.88, Table 7) and the external serum AUC was modest (0.67). The panel identified included known OSCC-related miRNAs (miR-21, miR-146b-5p) and a number of less studied candidate biomarkers whose function remains to be tested.

**Conclusion:**

The study demonstrates that a machine learning method can be applied to integrate public tissue miRNA datasets for identification of circulating miRNA biomarkers in OSCC. This is an exploratory study; the signature should not be considered as a validated serum test, but rather a tissue-derived candidate panel that offers limited transferability to serum samples and needs to be validated in more, larger, and independent circulating samples before it can be applied to a diagnostic purpose.

## Introduction

Oral squamous cell carcinoma (OSCC) is the most prevalent histopathological type of head and neck squamous cell carcinoma (HNSCC) and is a malignant tumor of the stratified squamous epithelium of the oral cavity, which makes up around 90% of all oral cavity cancers. The disease commonly involves the tongue, floor of the mouth, buccal mucosa, gingiva, hard palate, lip, and retromolar trigone, and is often preceded by oral potentially malignant disorders such as leukoplakia, erythroplakia, and oral submucous fibrosis [1]. GLOBOCAN 2020 showed that there were about 377,713 new cases of lip and oral cancer, and 177,757 deaths attributed to OSCC worldwide [2]. The disease burden is disproportionately high in South and Southeast Asian countries such as Pakistan and India which have the highest incidence and mortality rates and the 5-year survival of the disease is less than 50% due to late-stage diagnosis and limited oncology services [3].

The presentation of OSCC can vary from a white or red patch to a non-healing, ulcerated lesion with indurated margins, and advanced cases may be painful, with dysphagia, difficulty with tongue mobility, and cervical lymphadenopathy. At present, diagnostic methods have a low level of sensitivity for early lesions, and they are examiner-dependent, limited to small samples, and are invasive, leading to a high risk of diagnostic delay and poor survival [4]. The limitations highlight the critical need for the development of reliable, non-invasive, and affordable molecular markers that could be used to support early detection of OSCC in both specialist and community settings.

MicroRNAs (miRNAs) are small, single-stranded, endogenous, non-coding RNA molecules of about 19–25 nucleotides which are involved in almost all basic cellular functions such as proliferation, differentiation, apoptosis, and immune regulation [5]. In cancer, miRNAs can function as oncomiRs (miR-21 for example, which inhibits PTEN, PDCD4, and TPM1, promoting proliferation and chemoresistance) or as tumor suppressors (let-7 family, miR-34 for example, which inhibits oncogenic transcripts) [6, 7]. Circulating miRNAs released from tumor cells into blood, saliva, and other biofluids are remarkably stable because they are protected within extracellular vesicles or bound to RNA-binding proteins and lipoproteins, making them attractive candidates for non-invasive liquid biopsy in OSCC [8, 9]. Multiple studies have demonstrated that miRNAs derived from plasma, serum, or saliva, including exosomal cargoes, can be used to provide dynamic, real-time information on tumor behavior, as they decrease following tumor resection and increase with recurrence [10–14].

In spite of this promise, however, there are very few publicly available datasets of circulating miRNA in OSCC and most single study screens have relatively small sample sizes and are not statistically powerful enough to reliably identify candidate signatures across different cohorts [15, 16]. Public genomic repositories like the Gene Expression Omnibus (GEO) and The Cancer Genome Atlas (TCGA), combined with the uniformity of miRNA nomenclature in miRBase, represent a massive and diverse discovery reservoir that may be combined to produce reproducible biomarkers and to overcome sample size limitations [17–19]. The integration of expression data from several platforms, however, will introduce non-biological variability that needs to be corrected by batch-adjustment methods such as ComBat and by powerful meta-analytical frameworks [20, 21].

Machine learning (ML) has emerged as a key tool to identify reproducible classifiers from high-dimensional molecular datasets, and supervised learning approaches like logistic regression, support vector machines, random forest, and extreme gradient boosting (XGB) have been used to build models for the prediction of OSCC risk, modeling of prognosis, and miRNA-based diagnostics [22, 23]. To ensure that molecular classifiers are accurate, external validation is necessary on independent cohorts, and this has always been associated with overly optimistic performance [24]. In this context, the present study combines multiple GEO miRNA tissue datasets with the TCGA-HNSC oral-cavity miRNA dataset to drive a candidate signature, systematically compares five supervised ML algorithms, and tests the signature in an independent circulating serum dataset [25]. On these principles, and on the recent serum-based recurrence signature [26], the present study combines several GEO tissue datasets with the samples of the oral cavity from TCGA to build a stable miRNA signature, systematically compares five supervised ML algorithms and evaluates the resulting classifier in the largest publicly available independent circulating serum cohort (GSE113956).

The aim of this tissue-to-circulation biomarker discovery framework is to overcome the longstanding lack of blood-based miRNA cohorts that are relevant to OSCC and to promote the development of non-invasive diagnostic tools applicable in resource-limited environments.

## Methods

This study adopted a retrospective design by analyzing publicly available miRNA expression datasets of OSCC patients and non-cancer controls from GEO and TCGA-HNSC project. Seven GEO series (GSE113956, GSE45238, GSE28100, GSE168227, GSE98463, GSE103931, and GSE137865) together with oral-cavity samples from TCGA-HNSC were screened. The GSE113956 was reserved for use as an independent circulating serum validation set, and the rest of GEO and TCGA oral-cavity samples were used for tissue-based discovery. Human miRNA expression data with OSCC cases and non-cancer controls, adequate sample-level metadata with acceptable platform annotation to map probes to miRNA were included, whereas data from non-OSCC cancers, ambiguous labels, or failed quality control were excluded.

All analyses were performed in Python using GEOparse, requests, pandas, NumPy, SciPy, statsmodels, scikit-learn, xgboost, SHAP, matplotlib, and openpyxl, with a fixed random seed of 42 to ensure reproducibility. GEO expression matrices were parsed using GEOparse, probes were mapped to standardized miRNA identifiers via platform annotation and miRBase nomenclature and duplicate probes were summarized by the median. The miRNA expression data from TCGA-HNSC were downloaded from the Genomic Data Commons; samples of primary tumors from oral-cavity sites were designated as OSCC and solid tissue normal samples were designated as controls. The datasets were pre-processed individually, expression values were used log_2_ (*x* + 1)-transformed when needed, miRNAs that were present in less than 20% of the datasets were removed, and missing data were imputed using k-nearest-neighbor imputation, followed by quantile normalization within each dataset. MiRNAs that appeared in at least two independent datasets were retained and the individual matrices were aligned to the common harmonized miRNA set. ComBat or any other cross-dataset batch-correction method was not applied. Where necessary, each dataset was log2 transformed, imputed separately, and quantile normalized separately prior to merging together on the common miRNA set; effects of the datasets were not formally corrected, so leave-one-dataset-out validation reports the most conservative estimate of generalization.

Only exploratory visualization was done with principal component analysis. Differential expression was calculated using the two-sided Mann–Whitney *U* test and false discovery rate (FDR) was calculated to correct raw *p* values using Benjamini–Hochberg method. To avoid data leakage, feature selection was conducted independently in each of the five folds of the training set in a repeated stratified five-fold cross-validation (ten times), and the top 5, 10, 15, 20, and 30 miRNAs were tested. Five supervised binary classifiers (control = 0, OSCC = 1) were compared, logistic regression, linear support vector machine, radial-basis-function support vector machine, random forest, and XGBoost, each trained using balanced class weights.

Model performance was reported as accuracy, precision, sensitivity, F1-score, and mean receiver operating characteristic area under the curve (ROC-AUC) with standard deviation across folds. The signature was analyzed as continuous score, i.e., the model provides a predicted probability of OSCC for each sample and ROC-AUC was computed based on these probabilities. Accuracy, precision, sensitivity, and F1-score were computed using the default probability threshold of 0.5 (not optimized, nor validated separately) due to substantial variations in sensitivity across cohorts; no diagnostic threshold (cutoff) is proposed because of this variation. The stability of the feature selection was measured by the number of times a miRNA was selected across the folds of cross-validation and leave-one-dataset-out validation across tissue cohorts were performed to assess generalizability. The best-performing tissue-based model was retrained on all discovery datasets and externally validated on the independent circulating serum dataset (GSE113956) using accuracy, precision, sensitivity, F1-score, ROC-AUC, and confusion-matrix analysis, in line with recommended validation practices for molecular classifiers. Finally, the importance of miRNAs was summarized as mean absolute SHapley Additive exPlanations (SHAP) values derived from an independent XGBoost model. The final candidate panel was built by [25] a cross-validation-based selection frequency, differential expression, mean absolute SHAP importance, and consistent presence across multiple datasets.

## Results

### Dataset selection

**Fig. 1:**
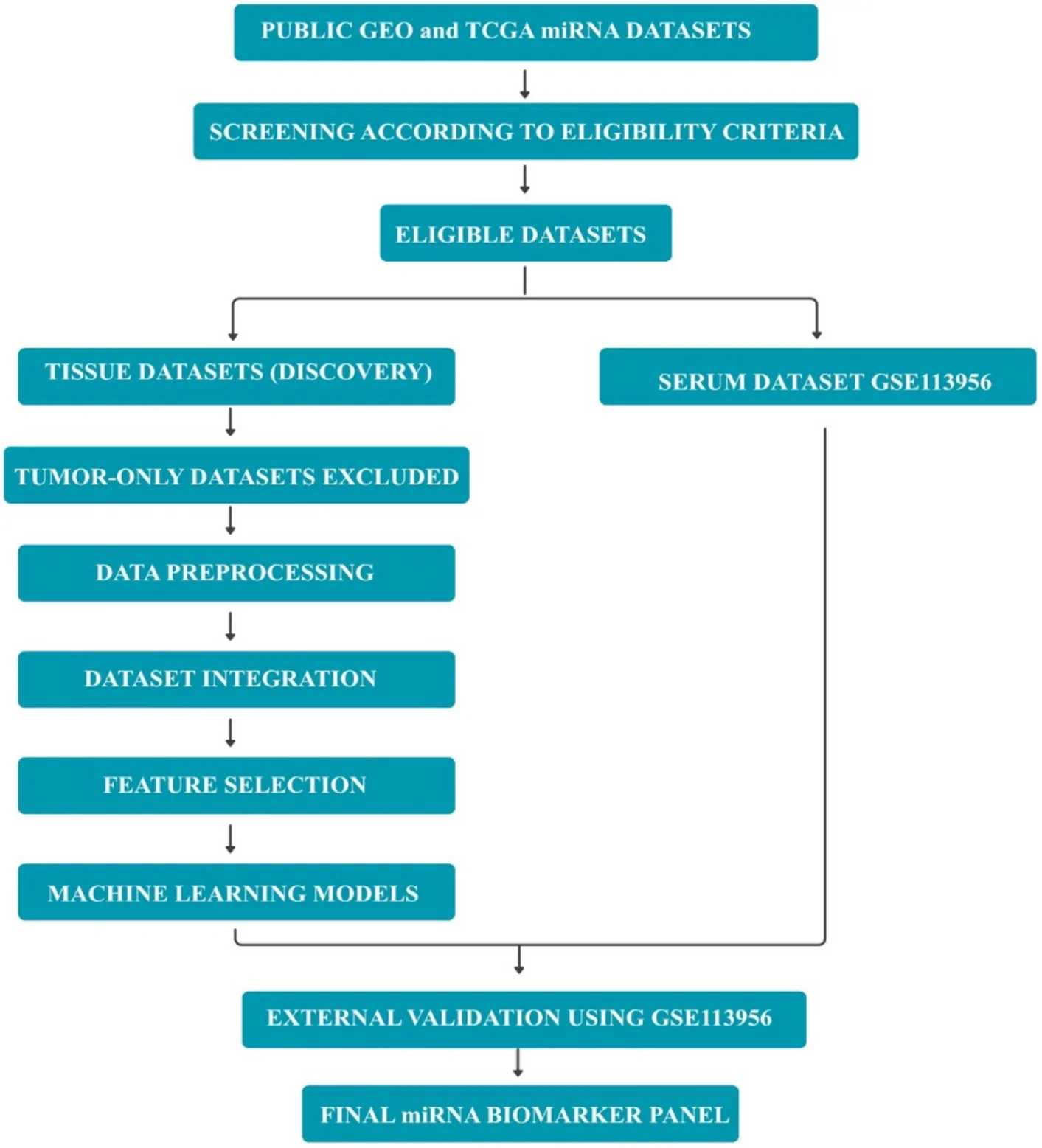
Dataset selection flowchart

Publicly available miRNA expression datasets related to OSCC were screened according to the pre-established eligibility criteria. Seven GEO datasets and oral cavity–related samples from TCGA-HNSC project fulfilled the selection criteria and were included in the analysis. Three datasets consisting exclusively of tumor samples without any control samples (GSE45238, GSE103931, and GSE137865) were excluded before model development, because batch correction and supervised model training cannot be performed unbiasedly on datasets that contain only one of the two classes. Only tissue datasets containing both OSCC and control samples (GSE28100, GSE168227, GSE98463, and TCGA-HNSC; 340 OSCC and 59 controls) were used for differential expression analysis, feature selection, model training, and SHAP analysis. The tissue-derived datasets were used for biomarker discovery and development of the ML models, while the circulating serum dataset (GSE113956) was only used for independent external validation (Fig. 1).

### Characteristics of datasets

The characteristics of datasets that are included, are summarized in Table 1. Included datasets represented different sample sizes and contained both OSCC and non-cancer control samples.

**Table 1:** Dataset characteristics and their role in this study

	Accession	Source	Samples	miRNAs	OSCC	Controls	Sample_type	Role
0	GSE113956	GEO	40	1960	25	15	Circulating_serum	External validation
1	GSE45238	GEO	40	858	40	0	Tissue	Excluded (tumor only)
2	GSE28100	GEO	20	939	17	3	Tissue	Model training
3	GSE168227	GEO	48	369	30	18	Tissue	Model training
4	GSE98463	GEO	16	32696	8	8	Tissue	Model training
5	GSE103931	GEO	30	232	30	0	Tissue	Excluded (tumor only)
6	GSE137865	GEO	18	1	18	0	Tissue	Excluded (tumor only)
7	TCGA_HNSC_ORAL	TCGA/GDC	315	1881	285	30	tissue_miRNA_seq	Model training

### Data preprocessing

After the data were retrieved, all datasets passed the data pre-processing pipeline (Table 2). Before integration, miRNA identifiers were normalized by platform, duplicate probes were merged, and expression matrices were normalized across platforms. As stated above, no cross-dataset batch correction (e.g., ComBat) was applied; normalization was performed separately within each dataset. The expression matrix was normalized and quality-controlled for the dataset, producing a single expression matrix that could be used for downstream statistical and machine learning analyses.

**Table 2:** Summary of preprocessing and integration of miRNA expression datasets

Preprocessing step	Outcome
miRNA name harmonization	Standardized across all datasets
Duplicate probe removal	Duplicate probes merged using median expression
Expression transformation	Log_2_ transformation applied where required
Missing value handling	Missing values imputed
Data normalization	Expression values normalized
Common miRNA selection	Shared miRNAs retained for integration
Dataset integration	Combined expression matrix generated

### Exploratory analysis

Two PCA plots were created to study clustering of the samples by disease status and dataset origin.

**Fig. 2:**
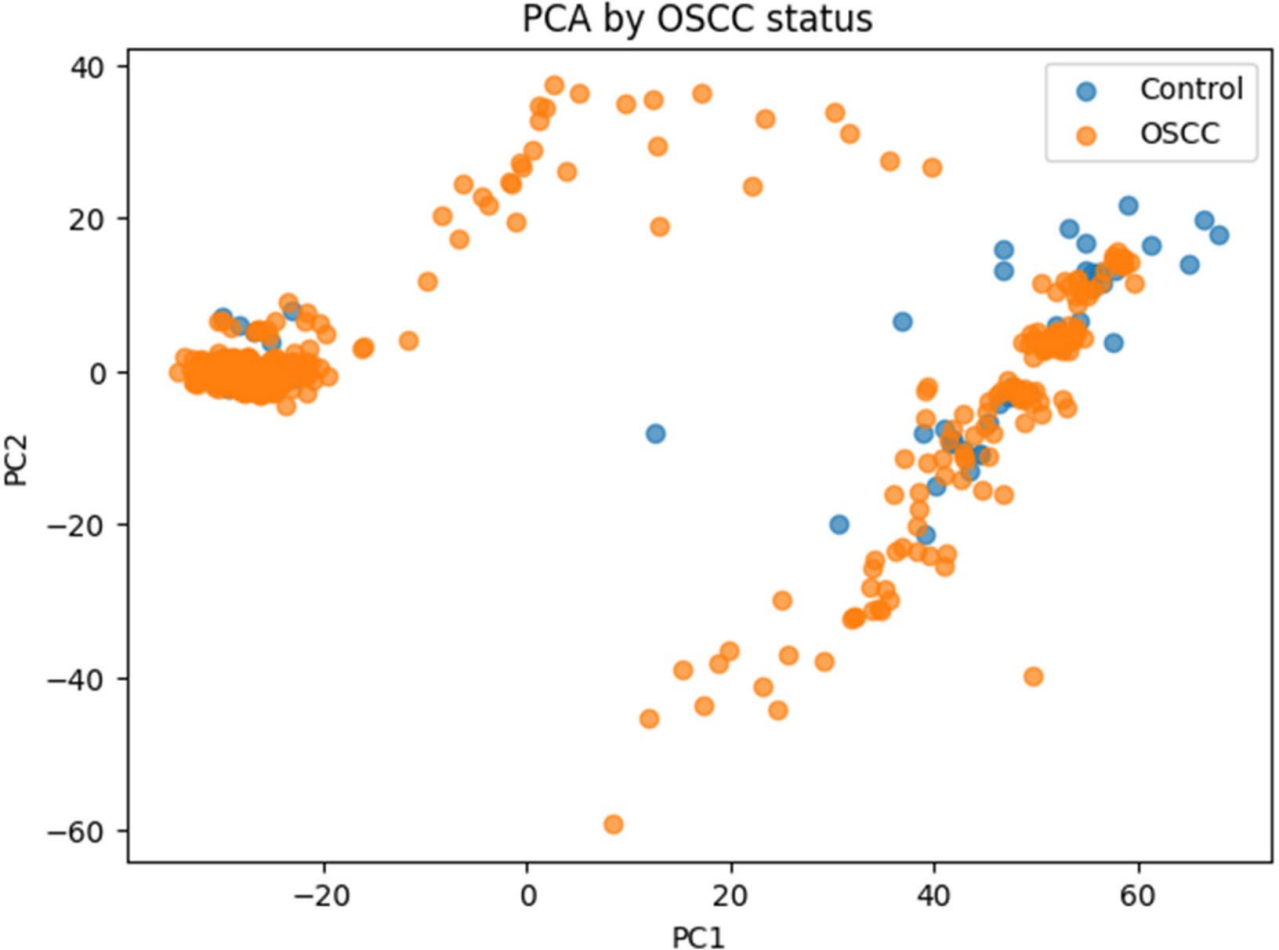
PCA of the integrated discovery cohort according to disease status

Figure 2 shows that samples are colored by disease status. There is some separation, but also overlap, between OSCC and control samples which suggests that they may be heterogeneous.

**Fig. 3:**
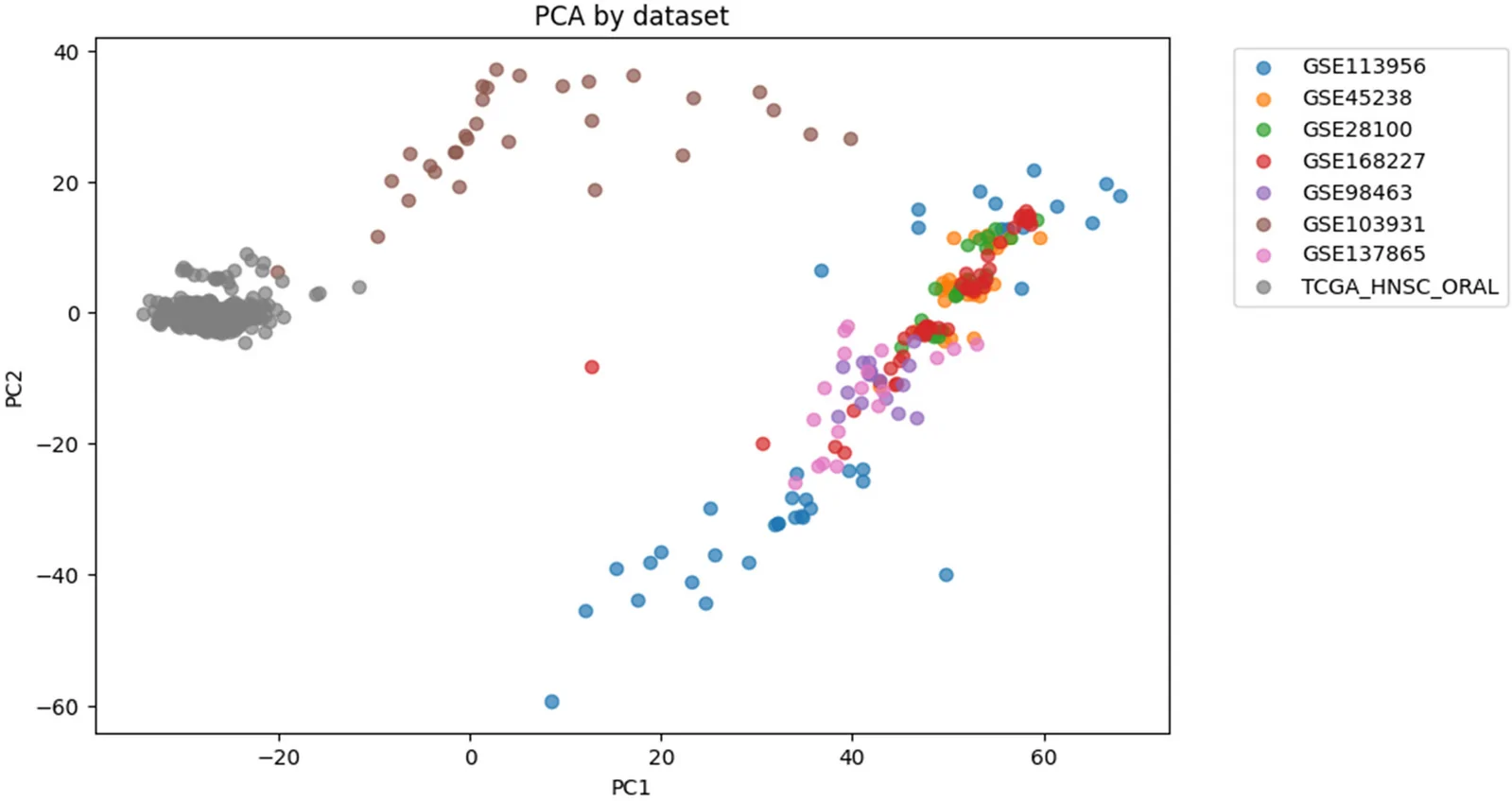
PCA of the integrated discovery cohort according to dataset source

Figure 3 shows the distribution of samples according to their dataset of origin. Some dataset-specific clustering is visible indicating that the source of the dataset further contributes to the overall variation.

### Differentially expressed miRNAs

A number of miRNAs were found to be differentially expressed between OSCC and control tissues. The candidate miRNAs were sorted by adjusted FDR, and the top features were kept for further machine learning analysis. The top differentially expressed miRNAs are presented in Table 3.

**Table 3:** Top differentially expressed miRNAs identified in the discovery cohort

	miRNA	log2FC_OSCC_vs_Control	p_value	FDR
1922	mir-4705	−3.499137	8.977442e−21	2.924850e−17
2735	mir-615	2.410751	5.180537e−19	5.626063e−16
472	mir-146b-5p	1.256925	4.473060e−19	5.626063e−16
252	mir-1251	−3.446908	2.249559e−18	1.832266e−15
2165	mir-488	−3.608498	3.228878e−18	2.103937e−15
809	mir-2681	−3.195235	1.388771e−17	7.541027e−15
418	mir-135a-1	−3.604339	2.513695e−17	1.169946e−14
3249	mir-99a	−1.253926	5.901606e−17	2.403429e−14
129	mir-100	−1.407765	1.113935e−16	4.032444e−14
2170	mir-490	−3.321535	1.526706e−16	4.974009e−14
896	mir-30a	−1.315078	3.863422e−16	1.144275e−13
2259	mir-5089	−3.084265	9.054576e−16	2.458317e−13
709	mir-21	1.473665	1.162780e−15	2.914106e−13
2848	mir-671	1.144177	1.712788e−15	3.985902e−13
136	mir-101-2	−1.214420	2.941558e−15	6.043041e−13
290	mir-1265	−2.766165	2.967731e−15	6.043041e−13
85	kshv-mir-k12-7	−0.839682	5.950185e−15	1.140335e−12
2174	mir-491-3p	0.682069	7.652586e−15	1.385118e−12
2003	mir-4740	−3.396813	2.758520e−14	4.730136e−12
419	mir-135a-2	−3.265773	3.086409e−14	5.027760e−12
1224	mir-3622a	−3.266201	4.956912e−14	7.690295e−12
909	mir-30c-2*	−0.435278	6.253763e−14	8.858591e−12
545	mir-181b-1	1.045361	6.032817e−14	8.858591e−12
639	mir-196b	1.141707	7.862208e−14	1.067295e−11
629	mir-195	−1.253825	9.776819e−14	1.274115e−11
1147	mir-337-5p	−0.703902	1.080917e−13	1.354472e−11
1391	mir-381	−1.355058	1.401590e−13	1.691252e−11
2278	mir-512-3p	−0.909191	1.467917e−13	1.694789e−11
860	mir-29c	−1.230291	1.508560e−13	1.694789e−11
689	mir-204	−1.530304	2.544251e−13	2.763057e−11

Asterisk denotes the passenger (star) strand of the miRNA duplex, following earlier miRBase nomenclature (mir-30c-2; currently designated miR-30c-2-3p)

### Feature selection

Feature selection was done to find out the optimal number of miRNAs for model evaluation. The repeated cross-validation was done to assess the candidate feature panel sizes, and their classification performance is reported in Table 4.

**Table 4:** Cross-validated performance of different feature panel sizes

	k_miRNAs	AUC_mean	AUC_SD	Accuracy	Sensitivity	F1
4	30	0.958962	0.022956	0.906266	0.924118	0.943489
3	20	0.956083	0.026477	0.901503	0.914118	0.940196
2	15	0.944104	0.033616	0.889728	0.900882	0.932511
1	10	0.937803	0.038569	0.872718	0.879706	0.920884
0	5	0.886259	0.056627	0.815329	0.820882	0.882410

The evaluated feature panel sizes demonstrated differences in predictive performance. The panel with the highest mean (30 miRNAs) ROC-AUC was chosen to be used in the development of the ML models.

### Machine learning model comparison

The predictive performance of the evaluated ML algorithms was compared using repeated cross-validation. Model performance was evaluated using accuracy, precision, recall, and F1-score. All classifiers evaluated are compared in terms of their performance in Table 5. Logistic regression achieved the highest mean ROC-AUC (0.959), whereas XGBoost and SVM-RBF achieved slightly higher accuracy (0.927 and 0.921, respectively, versus 0.906 for logistic regression).

**Table 5:** Performance comparison of machine learning models

	Model	AUC_mean	AUC_SD	Accuracy	Precision	Sensitivity	F1
0	Logistic regression	0.958962	0.022956	0.906266	0.964835	0.924118	0.943489
2	SVM-RBF	0.957821	0.030457	0.921060	0.960385	0.947059	0.953046
4	XGBoost	0.953106	0.024873	0.926826	0.936961	0.980588	0.958092
3	Random forest	0.950557	0.031011	0.913291	0.947231	0.951765	0.948897
1	SVM linear	0.946749	0.029571	0.902250	0.958594	0.925882	0.941416

### Stable miRNA signature

The stability of selected miRNAs was assessed based on the number of times that the miRNAs were carefully chosen (selection count) and the number of times that the miRNAs were selected (selection frequency) in repeated feature-selection iterations. MiRNAs that were chosen more frequently were considered more robust and reproducible biomarkers for distinguishing OSCC from control samples. The selection count and corresponding selection frequency of the identified miRNAs are presented in Table 6.

**Table 6:** Selection count and selection frequency of the stable miRNA signature

Index	miRNA	Selection_count	Selection_frequency
2	mir-4705	50	1.0
4	mir-135a-1	50	1.0
8	mir-2681	50	1.0
6	mir-146b-5p	50	1.0
5	mir-615	50	1.0
12	mir-1251	50	1.0
9	mir-488	50	1.0
0	mir-100	49	0.98
1	mir-99a	49	0.98
3	mir-30a	48	0.96
19	mir-671	48	0.96
16	mir-490	47	0.94
7	mir-21	47	0.94
10	mir-5089	46	0.92
11	mir-101-2	44	0.88
31	mir-1265	42	0.84
17	mir-491-3p	41	0.82
30	kshv-mir-k12-7	41	0.82
13	mir-4740	38	0.76
54	mir-135a-2	33	0.66
52	mir-181b-1	29	0.58
18	mir-196b	28	0.56
25	mir-337-5p	28	0.56
22	mir-195	27	0.54
26	mir-3622a	27	0.54
35	mir-30c-2*	25	0.5
15	mir-381	22	0.44
24	mir-1307	21	0.42
14	mir-29c	20	0.4
65	mir-512-3p	20	0.4

Asterisk denotes the passenger (star) strand of the miRNA duplex, following earlier miRBase nomenclature (mir-30c-2; currently designated miR-30c-2-3p)

The identified miRNAs showed different degrees of selection stability in repeated cross-validations. MiRNAs with the highest selection counts and selection frequencies were consistently retained during feature selection, indicating greater robustness and reproducibility. These stable miRNAs were then selected for further modeling and subsequent development of the final candidate miRNA biomarker panel.

### Leave-one-dataset-out validation

To estimate the robustness and generalizability of selected machine learning model, leave-one-dataset-out validation was performed. For each iteration, one full tissue dataset was withheld from the training set and treated as an independent testing set, and the other tissue datasets were used for model development. The performance obtained for each held-out dataset is in Table 7.

**Table 7:** Leave-one-dataset-out validation performance of the final machine learning model

Held_out_dataset	Test_Samples	AUC	Accuracy	Precision	Sensitivity	F1
GSE168227	48	0.542593	0.520833	0.600000	0.700000	0.646154
GSE28100	20	0.882353	0.650000	1.000000	0.588235	0.740741
GSE98463	16	0.859375	0.687500	0.636364	0.875000	0.736842
TCGA_HNSC_ORAL	315	0.814971	0.231746	1.000000	0.150877	0.262195

The leave-one-dataset-out validation confirmed large variations in performance across the tissue sets left out of the training set (AUC range 0.54–0.88). When TCGA-HNSC was held out, the AUC for TCGA remained 0.81 while sensitivity fell to 0.15. This is consistent with TCGA contributing 285 of the 340 OSCC samples (84%) and 30 of the 59 controls (51%) in the training data, so that holding it out leaves only 84 samples (29 controls) for training. This pattern implies that the ranking of the samples is partially carried over between the cohorts and that the threshold of decision is not carried over. Cautious generalizability of the classifier is therefore interpreted, and this wide variation reported as a limitation.

### External validation using circulating dataset

In the step of tissue-based model development, the logistic regression model was determined as the best one and was validated by the independent circulating serum miRNA dataset (GSE113956). This is an external validation (Table 8) that evaluated the discriminating capacity of the tissue-based miRNA signature between OSCC cases and healthy controls in an external circulating cohort.

**Table 8:** Performance of the logistic regression model on the independent circulating miRNA validation dataset

	Precision	Recall	F1-Score	Support
Control	0.67	0.53	0.59	15
OSCC	0.75	0.84	0.79	25
Accuracy			0.72	40
Macro avg	0.71	0.69	0.69	40
Weighted avg	0.72	0.72	0.72	40

**Fig. 4:**
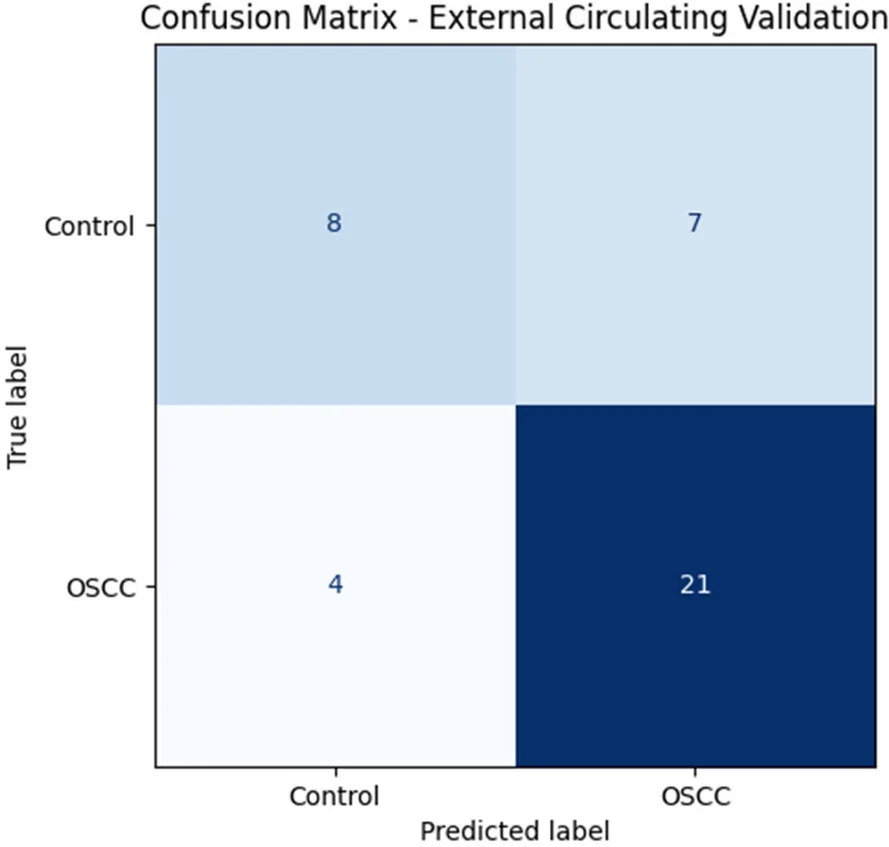
Confusion matrix of the logistic regression model for independent circulating miRNA validation dataset External circulating validation AUC:0.6746666666666666

**Fig. 5:**
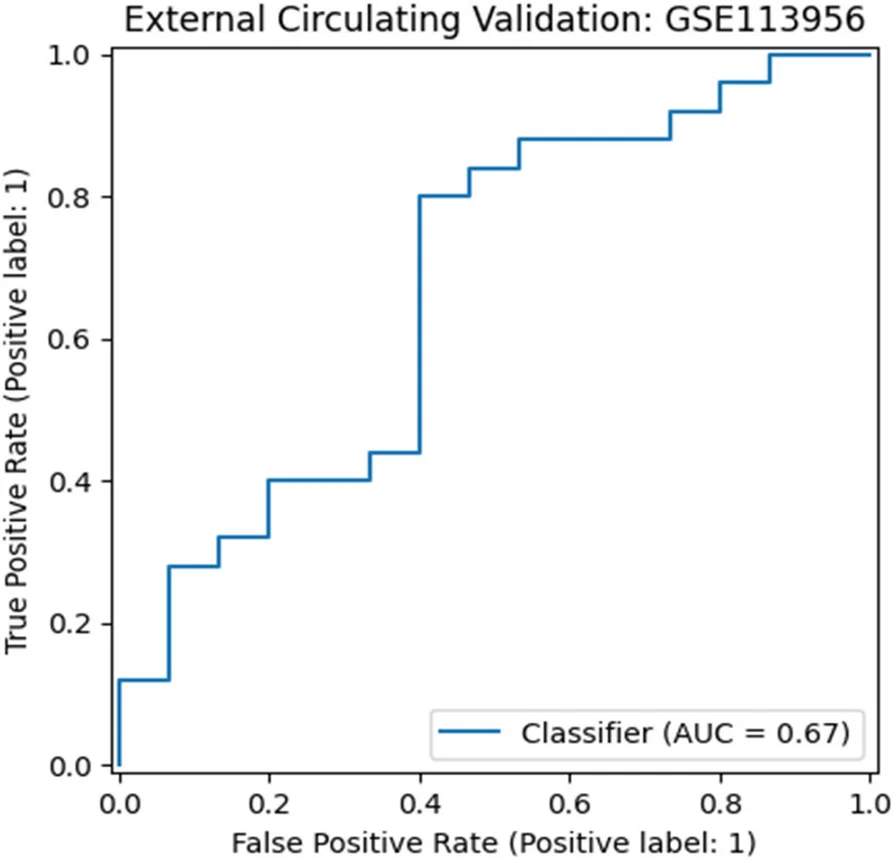
ROC curve of the logistic regression model for independent circulating miRNA validation dataset

The logistic regression model achieved an external AUC of 0.67 on the independent circulating miRNA dataset, with modest discriminatory performance. The corresponding accuracy, precision, recall, and F1-score are presented in Table 8, while the confusion matrix and ROC curve are shown in Figs. 4 and 5, respectively. The results presented here suggest that the tissue signature of miRNAs is only partially represented in the circulating compartment and that it should not be regarded as a validated serum marker.

### SHAP analysis

SHAP analysis was performed to investigate the contribution of individual miRNAs to the final machine learning model. The importance of each feature was quantified using the mean absolute SHAP value.

**Fig. 6:**
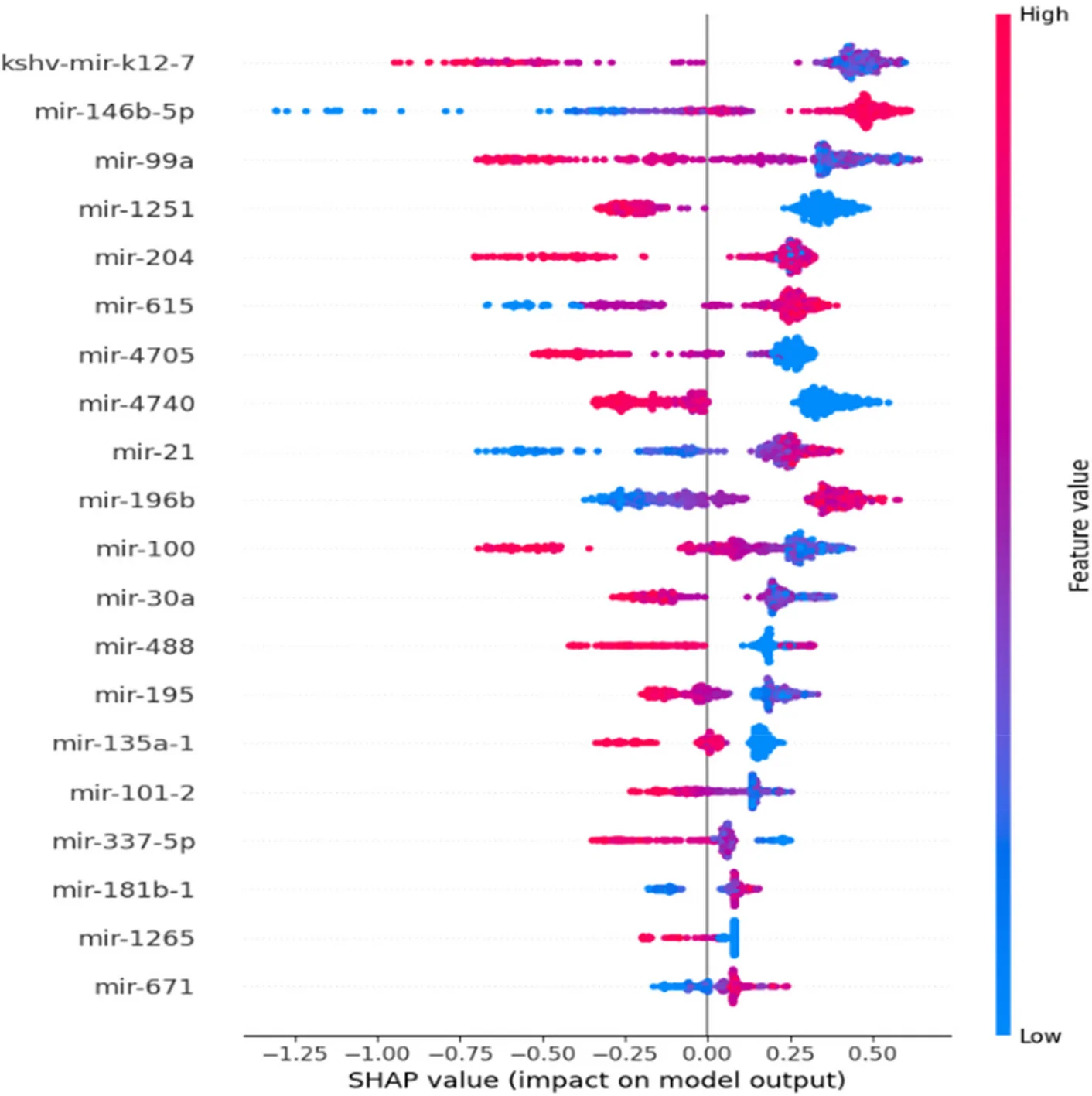
SHAP summary plot illustrating the contribution of the selected miRNAs to the prediction of the final logistic regression model

Several miRNAs demonstrated consistently greater contributions to model prediction than the remaining features (Fig. 6 and Table 9).

**Table 9:** Ranking of the selected miRNAs according to mean absolute SHAP values in the final logistic regression model

	miRNA	mean_abs_SHAP
16	kshv-mir-k12-7	0.482718
2	mir-146b-5p	0.406943
7	mir-99a	0.362317
3	mir-1251	0.314508
29	mir-204	0.295633
1	mir-615	0.276183
0	mir-4705	0.272447
19	mir-4740	0.270089
12	mir-21	0.257113
23	mir-196b	0.255998
8	mir-100	0.253071
10	mir-30a	0.201861
4	mir-488	0.194008
24	mir-195	0.136536
6	mir-135a-1	0.125154
15	mir-101-2	0.111105
25	mir-337-5p	0.098199
22	mir-181b-1	0.097961
14	mir-1265	0.082832
13	mir-671	0.068057

### Final candidate miRNA panel

The final candidate miRNA panel (Table 10) was selected by taking into consideration the evidence from the differential expression analysis, feature stability, SHAP feature importance, and machine learning performance. The miRNAs that were commonly detected by these complementary analyses were selected as the final candidate biomarkers.

**Table 10:** Final candidate miRNA panel

	miRNA	Selection_count	selection_frequency	log2FC_OSCC_vs_Control	p_value	FDR	mean_abs_SHAP	number_of_datasets_present
3	mir-146b-5p	50	1.00	1.256925	4.473060e−19	5.626063e−16	0.406943	5
5	mir-1251	50	1.00	−3.446908	2.249559e−18	1.832266e−15	0.314508	5
4	mir-615	50	1.00	2.410751	5.180537e−19	5.626063e−16	0.276183	2
0	mir-4705	50	1.00	−3.499137	8.977442e−21	2.924850e−17	0.272447	3
6	mir-488	50	1.00	−3.608498	3.228878e−18	2.103937e−15	0.194008	4
1	mir-135a-1	50	1.00	−3.604339	2.513695e−17	1.169946e−14	0.125154	2
2	mir-2681	50	1.00	−3.195235	1.388771e−17	7.541027e−15	0.056546	2
8	mir-99a	49	0.98	−1.253926	5.901606e−17	2.403429e−14	0.362317	6
7	mir-100	49	0.98	−1.407765	1.113935e−16	4.032444e−14	0.253071	6
9	mir-30a	48	0.96	−1.315078	3.863422e−16	1.144275e−13	0.201861	6
10	mir-671	48	0.96	1.144177	1.712788e−15	3.985902e−13	0.068057	2
12	mir-21	47	0.94	1.473665	1.162780e−15	2.914106e−13	0.257113	6
11	mir-490	47	0.94	−3.321535	1.526706e−16	4.974009e−14	0.002625	2
13	mir-5089	46	0.92	−3.084265	9.054576e−16	2.458317e−13	0.065003	3
14	mir-101-2	44	0.88	−1.214420	2.941558e−15	6.043041e−13	0.111105	2
15	mir-1265	42	0.84	−2.766165	2.967731e−15	6.043041e−13	0.082832	5
17	kshv-mir-k12-7	41	0.82	−0.839682	5.950185e−15	1.140335e−12	0.482718	2
16	mir-491-3p	41	0.82	0.682069	7.652586e−15	1.385118e−12	0.033475	5
18	mir-4740	38	0.76	−3.396813	2.758520e−14	4.730136e−12	0.270089	2
19	mir-135a-2	33	0.66	−3.265773	3.086409e−14	5.027760e−12	0.035374	2
20	mir-181b-1	29	0.58	1.045361	6.032817e−14	8.858591e−12	0.097961	2
21	mir-196b	28	0.56	1.141707	7.862208e−14	1.067295e−11	0.255998	5
22	mir-337-5p	28	0.56	−0.703902	1.080917e−13	1.354472e−11	0.098199	5
23	mir-195	27	0.54	−1.253825	9.776819e−14	1.274115e−11	0.136536	6
24	mir-3622a	27	0.54	−3.266201	4.956912e−14	7.690295e−12	0.017792	2
25	mir-30c-2*	25	0.50	−0.435278	6.253763e−14	8.858591e−12	0.043510	3
26	mir-381	22	0.44	−1.355058	1.401590e−13	1.691252e−11	0.049040	7
27	mir-1307	21	0.42	1.136797	3.671304e−13	3.751396e−11	NaN	4
29	mir-512-3p	20	0.40	−0.909191	1.467917e−13	1.694789e−11	0.009887	5
28	mir-29c	20	0.40	−1.230291	1.508560e−13	1.694789e−11	0.008334	6

Asterisk denotes the passenger (star) strand of the miRNA duplex, following earlier miRBase nomenclature (mir-30c-2; currently designated miR-30c-2-3p)

These miRNAs are a candidate miRNA panel for further validation studies, and not yet a clinically validated miRNA signature.

## Discussion

To fill one of the most persistent challenges in the circulating miRNA biomarker arena for OSCC, namely the lack of sufficiently powered circulating cohorts, this study explored the significantly larger tissue transcriptomic resource of publicly available data as a discovery cohort and then tested the discovered signature in circulation. To create a harmonized discovery matrix for multiple GEO tissue datasets, the datasets were combined with the TCGA-HNSC head and neck cohort, measuring differential expression among biologically diverse OSCC samples [17, 19, 21]. From this integrated resource, a candidate panel of 30 miRNAs was identified, and five supervised machine learning classifiers were trained and compared, with logistic regression showing the most stable behavior, and went on to test an external circulating serum miRNA panel on an independent serum sample set, producing an AUC of 0.67 [15, 16]. We used tissue for discovery because no large publicly available circulating miRNA datasets for OSCC exist, and GSE113956, the only publicly available serum dataset that we found, was intentionally omitted as a test of whether a tissue-derived signal is translatable into blood. It has a partial carry over of 0.67 (external AUC). The 30 miRNAs should thus be considered as tissue-derived candidate biomarkers that have the potential to be found in the circulation, but not as a validated serum test. This design assumption has restricted the use of the panel for immediate diagnosis and should be considered when interpreting our results.

Several factors likely contribute to this tissue-to-serum performance gap: differences in the cell of origin of circulating miRNAs, differences in profiling platforms between the tissue datasets and the serum dataset, the small size of the serum cohort (25 OSCC, 15 controls), and differences in normalization strategy. For a direct test of the transferability of the samples, matched tissue and serum from the same patients will be required.

The canonical oncomiR, miR-21 is one consistently upregulated in OSCC tissue, saliva, and serum, and it binds to the p53 pathway and tumor suppressors, including PTEN and PDCD4, to promote proliferation and inhibit apoptosis; its frequent presence in circulating cohorts validates its inclusion in the present signature [6, 7]. miR-146b-5p, another panel member, has likewise been linked to OSCC in previous studies. The panel also includes the tumor suppressive miRNAs miR-99a and miR-100, which are downregulated in signals, thus removing a brake on the translation axis of PI3K/AKT/mTOR, and linked to aggressive tumor behavior in TCGA-derived signals. Some other members of the miR-30 family that were previously described in OSCC tissue and plasma classifiers, as well as less frequently reported miRNAs such as miR-1251, miR-4705, miR-2681, and the viral-associated kshv-miR-K12-7 could only be detected because the discovery cohort was large enough to provide statistical visibility for the miRNAs that are under-represented in single-study analyses and that only became reliably annotated in more recent miRBase releases [15, 18, 22]. This panel thus includes some known miRNAs associated with OSCC, as well as other less studied candidates that need to be tested. We did not perform target prediction or pathway enrichment analysis, because the aim of this study was to build and test a classifier rather than to characterize miRNA function; target and pathway analysis, followed by experimental validation, is a necessary next step.

The overall methodology of the present study is consistent with the larger trend of the use of ML in oncology, where the supervised learning algorithms are well established for deriving meaningful classifiers from high-dimensional and correlated molecular data [22, 23]. In the present setting, the signal was distributed among a large number of miRNAs with approximately additive contributions, and penalized linear models generally outperformed flexible non-linear learners like support vector machines, random forest, and XGBoost; this is likely because a miRNA expression matrix contains hundreds of features that are correlated and measured on relatively few samples. We deliberately used classical machine learning methods rather than deep learning for three reasons. This is a very unbalanced dataset of tissue discovery (59 controls vs. 340 OSCC samples) and deep models tend to overfit when the number of samples is small compared to the number of features of the misRNAs. Second, we aimed for a small, interpretable panel that penalized linear models as well as SHAP-based interpretation support. Third, the linear model was already competitive with the more flexible models (Table 5) and so the more complex the model, the worse it did in this scenario. The moderate but biologically relevant AUC of 0.67 is also because the classifier was evaluated in different biological and technical compartments, discovery in tissue, and validation in serum; and internal cross-validation has been known to overestimate real-world performance [24]. This means that the serum miRNA pool is subject to active release from extracellular vesicles, passive release from apoptotic and necrotic cells, and binding to Argonaute and lipoprotein carriers, making the serum miRNA profile a partial and biased representation of the tumor [8–10, 25]. Inter-study variability is further due to profiling platforms, RNA extraction protocols, and normalization strategies, and even well-designed circulating OSCC studies have reported similar results of classifiers being attenuated when transferred between different cohorts [11, 12, 14, 16, 25]. Consistent with this, a serum-based miRNA signature was derived that detects recurrent OSCC before it is clinically evident [26]; their signature was derived directly from serum, whereas ours was derived from tissue and showed only partial transferability to serum, underscoring that tissue-derived and serum-derived signatures capture overlapping but distinct signals.

These findings are strengthened by a number of methodological strengths. The discovery matrix was sourced from a number of public repositories and was much larger and more diverse than any single-institution collection, which decreases the risk of overfitting a single population or platform [15, 17]. The results from external validation on an independent circulating dataset directly address the issue of optimistic reporting in the development of molecular classifiers [24], and the systematic comparison of five ML models gives a more balanced account of algorithm behavior than a study of only one algorithm [22, 23]. However, SHAP-based interpretation provides more transparency by decomposing the classifier into contributions to the model output made by each individual feature, which can be evaluated on a biological level and compared across different features, making it not only a statistical object but also a set of miRNAs, whose individual contribution to model output can be traced and cross-referenced with the known biological context of OSCC.

## Limitations

This study has several limitations. First, there was no overlap between the tissue matrix datasets and the circulating serum miRNA datasets; the AUC for external validation was not very high, suggesting that the panel cannot be viewed as diagnostic, but rather as explorative and hypothesis-generating. Second, there was no formal cross-dataset batch correction (no ComBat): the datasets were normalized individually and then merged on common miRNAs; therefore, no formal batch correction of the datasets was performed; the leave-one-dataset-out validation (Table 7) had a large range of AUC (0.54–0.88) values and is thus the most conservative estimate of generalization. Third, all the datasets included here have only samples that are not cancerous or otherwise abnormal; the signature has not been tested against samples of benign or premalignant oral lesions and testing in cohorts of such lesions is necessary. Fourth, most of the public datasets used have no consistent clinical information (stage, tumor site, smoking, age, sex), so we could not adjust for these clinical covariates across the pooled data; future work should test the panel in cohorts with full clinical annotation. Fifth, we did not perform target, pathway, or other functional analysis of the panel. Sixth, to our knowledge, GSE113956 is the only publicly available serum or plasma miRNA dataset with both OSCC cases and controls (we searched GEO, ArrayExpress, and SRA); it is small (25 OSCC, 15 controls) and its miRNA levels were measured with a hybridization-based platform, which has been shown to be less accurate than RT-qPCR- or sequencing-based methods. Further validation is thus necessary in larger, prospective, and independent cohorts in circulation, which can be done by RT-qPCR or sequencing.

## Conclusion

This exploratory study shows that an integrative computational approach, discovery across multiple public GEO tissue datasets and the TCGA-HNSC oral-cavity cohort, followed by testing in an independent circulating serum cohort, can generate a candidate miRNA panel for OSCC. A set of 30 candidate miRNAs was identified, including well-known OSCC-related molecules such as miR-21, miR-146b-5p, miR-99a, miR-100, miR-195, and miR-29c as well as less frequently reported and more recently annotated miRNAs. A tissue-derived miRNA signature brings a limited diagnostic signal to the blood, as demonstrated by the reasonable external AUC of 0.67 obtained with the logistic regression model on serum. These findings are hypothesis-generating as larger independent, prospective cohorts of circulating samples (ideally matched tissue and serum with both RT-qPCR and sequencing) are required to assess any diagnostic application of this panel.

## Data Availability

The datasets analyzed in this study are publicly available. Gene Expression Omnibus (GEO) datasets can be accessed through the NCBI GEO database, and The Cancer Genome Atlas (TCGA) data can be accessed through the Genomic Data Commons (GDC) Data Portal. The accession numbers of the GEO datasets used in this study are provided in the manuscript. All data generated or analyzed during this study are included in the published article where applicable.
